# The prediction models for postoperative overall survival and disease‐free survival in patients with breast cancer

**DOI:** 10.1002/cam4.1092

**Published:** 2017-05-24

**Authors:** Daichi Shigemizu, Takuji Iwase, Masataka Yoshimoto, Yasuyo Suzuki, Fuyuki Miya, Keith A Boroevich, Toyomasa Katagiri, Hitoshi Zembutsu, Tatsuhiko Tsunoda

**Affiliations:** ^1^ Department of Medical Science Mathematics Medical Research Institute Tokyo Medical and Dental University Tokyo Japan; ^2^ Laboratory for Medical Science Mathematics RIKEN Center for Integrative Medical Sciences Yokohama Japan; ^3^ CREST Japan Science and Technology Agency Tokyo Japan; ^4^ Department of Breast Surgical Oncology Breast Oncology Center Cancer Institute Hospital Japanese Foundation for Cancer Research Tokyo Japan; ^5^ Yoshimoto Breast Clinic Tokyo Japan; ^6^ First Department of Surgery Sapporo Medical University School of Medicine Sapporo Japan; ^7^ Division of Genome Medicine Institute for Genome Research Tokushima University Tokushima Japan; ^8^ Human Genome Center Institute of Medical Science The University of Tokyo Tokyo Japan; ^9^ Division of Genetics National Cancer Center Research Institute Tokyo Japan; ^10^ Department for Medical Genome Sciences Medical Genome Center National Center for Geriatrics and Gerontology Obu Japan

**Keywords:** Breast cancer, disease‐free survival, MammaPrint genes, overall survival, prediction model

## Abstract

The goal of this study is to establish a method for predicting overall survival (OS) and disease‐free survival (DFS) in breast cancer patients after surgical operation. The gene expression profiles of cancer tissues from the patients, who underwent complete surgical resection of breast cancer and were subsequently monitored for postoperative survival, were analyzed using cDNA microarrays. We detected seven and three probes/genes associated with the postoperative OS and DFS, respectively, from our discovery cohort data. By incorporating these genes associated with the postoperative survival into MammaPrint genes, often used to predict prognosis of patients with early‐stage breast cancer, we constructed postoperative OS and DFS prediction models from the discovery cohort data using a Cox proportional hazard model. The predictive ability of the models was evaluated in another independent cohort using Kaplan–Meier (KM) curves and the area under the receiver operating characteristic curve (AUC). The KM curves showed a statistically significant difference between the predicted high‐ and low‐risk groups in both OS (log‐rank trend test *P* = 0.0033) and DFS (log‐rank trend test *P* = 0.00030). The models also achieved high AUC scores of 0.71 in OS and of 0.60 in DFS. Furthermore, our models had improved KM curves when compared to the models using MammaPrint genes (OS:* P* = 0.0058, DFS:* P* = 0.00054). Similar results were observed when our model was tested in publicly available datasets. These observations indicate that there is still room for improvement in the current methods of predicting postoperative OS and DFS in breast cancer.

## Introduction

Breast cancer was the most frequently diagnosed cancer in women in 2015 1, 2, 3. An estimated 231,840 women were expected to be diagnosed with the breast cancer in the United States 1, 4. Although the survival rate is improving, breast cancer is still the second major cause of cancer‐related death in women 3, 5, largely due to the invasive nature and the high rate of metastasis in breast cancers 6. If cancer spreads to the lymph nodes around the clavicle or to the other organs (distant stage), the overall median survival is low, ranging from 15–27 months 7, 8, 9. On the other hand, if the cancer has not spread to lymph nodes (early stage), the overall survival increases substantially 1. Therefore, early detection of the cancer plays an important role in extending a patient's life expectancy.

Currently, surgery is the primary treatment for breast cancer without metastasis 4. Either breast‐conserving surgery (surgical removal of the tumor and surrounding tissue) or mastectomy (surgical removal of the breast) is performed 10. However, even in cases of complete surgical resection, postoperative overall survival (OS) and disease‐free survival (DFS) can vary greatly among patients 11, 12. Previous studies have shown that breast cancer can be classified into subclasses based on their gene expression profiles 13, 14, 15, 16, and highly significant differences in OS and DFS can be observed between these subclasses 14. Sorlie et al. reported that a basal group of breast cancer patients categorized by expression of genes associated with myoepithelial cells, *KRT5* (keratin 5), *KRT17* (keratin 17), *CNN1* (calponin 1), *CAV1* (caveolin), and *LAMB1* (laminin), is most aggressive with a poor DFS and OS 14. In addition, several genetic tests using gene expression profiles have been developed to predict clinical outcomes 17, 18. As one of the tests, MammaPrint 19 was developed as a diagnostic tool to predict prognosis of breast cancer for OS and DFS using the expression data for 70 genes 20. However, to our knowledge, the analyses of the subsequent improved prediction models have yet to be reported.

Here, we identified genes associated with the postoperative OS and DFS from our gene expression data of breast cancer patients, who underwent complete surgical resection and whose subsequent postoperative survival was recorded. By incorporating the genes associated with the postoperative OS and DFS, as well as five genes associated with a poor DFS and OS reported by Sorlie et al. 14, into the MammaPrint gene set 21, we constructed prediction models from our discovery cohort using a Cox proportional hazard model and investigated whether the models were improved. The predictive ability of our models was evaluated in several independent cohorts using both Kaplan–Meier (KM) curves and the area under the receiver operating characteristic curve (AUC). Not only were both of our models for OS and DFS validated in the independent test set, but our models also showed improved KM curves when compared with those using the MammaPrint gene set.

## Materials and Methods

### Study subjects

In the first discovery cohort, primary breast cancers were obtained with informed consent from 81 patients who were treated at the Department of Breast Surgery, Cancer Institute Hospital of Japanese Foundation for Cancer Research, Tokyo (Table S1). Clinical information was obtained from medical records as previously reported 22. In the second discovery cohort, breast cancer tissue samples from core needle biopsy or surgical biopsy and corresponding clinical information was obtained from three hospitals (The Cancer Institute Hospital of JFCR, Sapporo Breast Surgery Clinic, and Sapporo Medical University) after each patient had provided informed consent 23. A total of 16 cancer samples that had been confirmed histologically as invasive breast cancer were used in the second discovery cohort (Table S1). A piece of cancer tissue was taken from each patient at the time of biopsy, before chemotherapy, as described previously 23. The samples used in the first and second discovery cohorts were immediately embedded in TissueTek OCT compound (Sakura, Tokyo, Japan), frozen, and stored at −80°C. The frozen tissues were sliced into 8 *μ*m sections using a cryostat (Sakura) and then stained with H&E for histological examination. Breast cancer cells were selectively enriched for our experiments using the EZ cut system with a pulsed UV narrow beam focus laser (SL Microtest GmbH, Germany) according to the manufacturer's protocols. A mixture of RNA isolated from normal breast ductal cells of breast cancer patients served as a normal control as described previously 22, 23.

Validation cohorts were obtained from the GEO (accession GSE42568 24 and GSE1456 25) and TCGA (https://cancergenome.nih.gov/) databases. The GSE42568 cohort has 104 patients with breast cancer, of which 48 patients experienced relapse and 35 patients died. The GSE1456 cohort has 159 patients with breast cancer, of which 40 patients experienced relapse and 29 patients died. These cohorts were used as validation sets for both the OS and DFS prediction models. One gene in the MammaPrint gene set, *PALM2*, was excluded from the GSE1456 validation analysis due to low signal intensities and missing values. The TCGA cohort has 1090 breast cancer patients with RNA‐seq and clinical information, of which 151 patients died. As there was no information regarding relapse, this cohort was used as a validation set for only the OS prediction models. Six genes (*QSCN6L1*,* KNTC2*,* ORC6L*,* GPR126*,* PECI*, and *ZNF533*) in the MammaPrint gene set were excluded from the TCGA validation analysis due to low signal intensities and missing values.

### Microarray gene expression data

Microarray experiments were performed as previously reported 22, 23. We used mixture of RNAs isolated from normal breast ductal cells of breast cancer patients as universal reference RNA (URR) for the microarray experiments. Briefly, cDNAs derived from breast cancer cells and URR were labeled with Cy5 and Cy3, respectively. Equal amounts (2.5 *μ*g for each microarray slide) of Cy5‐ and Cy3‐labeled cDNA were combined, and then the mixed cDNA was hybridized for 14–16 h at 42°C on microarray composed of six glass slides with 27,648 cDNA probes. After hybridization, the microarrays were scanned and the fluorescence signals were digitalized using the Array Scanner Generation III (Amersham) and ArrayVision computer program (Amersham), as described previously 26.

To correct for bias between microarrays, quantile normalization 27 was applied to all microarray data for each Cy and slide set using R software. The signal ratio of each probe was calculated according to the following procedure. (1) If the both Cy5 and Cy3 signal of a probe were less than 10,000, the ratio was handled as missing value. (2) If only one of either Cy3 or Cy5 signal was less than 10,000, the lower signal was converted to 10,000. (3) The ratio was calculated by the Cy5/Cy3 signal intensity. Next, the log_2_‐transformed ratio was converted to a *z*‐score for each microarray slide, the *z*‐score conversion was performed using following formula:

$$z=(j_{i}-j^{{}^{-}})/\sigma$$

where *z* is the *z*‐score for probe *j*_*i*_, *j*_*i*_ is log_2_‐transformed ratio of a probe, $\overset{¯}{J}$ is mean ratio for log_2_‐transformed ratio of all probes in the microarray slide, and *σ* is the standard deviation from the mean. In addition, these data were further normalized per gene in each dataset by the *z* transformation for cross‐platform analysis.

## Statistical analysis

Both OS and DFS were investigated using Cox proportional hazard models with “time since diagnosis” as the underlying time scale. We first performed gene selection through the following steps: (1) The most significantly differentially expressed genes under different conditions (OS: survival and dead, DFS: relapse and nonrelapse) using a Cox proportional hazard model from the first discovery cohort data (*P* < 0.0003). (2) Genes showing effects in the same direction as genes detected in step 1 from the second discovery cohort data. (3) Genes showing *q* < 0.2 in the combined discovery cohort data. The time since diagnosis for OS was then calculated from the date of postoperative check up to the date of death or the follow‐up cutoff, and that for DFS was calculated from the date of postoperative check up to the date of distant organ relapse.

Using genes determined under three selection conditions as described above, we calculated a prognostic index for each sample as defined by

$$\text{prognostic index}=\sum_{i}\beta_{i}\times X_{i}$$

where *β*_*i*_ is the estimated regression coefficient of each gene using a Cox proportional hazard model in the combined discovery cohort data, and *X*_*i*_ is the *z*‐transformed score of the gene. The prognostic index was calculated in each sample of the combined discovery cohort. We classified samples into two groups (high‐ and low‐risk groups) by an optimal cutoff value of the prognostic index 28. The optimal cutoff value indicated a minimum log‐rank trend test *P*‐value by comparing differences between high‐ and low‐risk groups in the combined discovery cohort in OS and DFS, respectively. These optimal cutoff values were used for the validation analysis of our OS and DFS prediction models.

KM curves were constructed to illustrate difference in survival in OS and DFS. The log‐rank test was used to compare the different conditions. A *P*‐value of 0.05 or less was considered statistically significant. Moreover, the receiver operator characteristic (ROC) curves 29 and the area under the curve (AUC) were indicated as the discriminative accuracy of these survival prediction models. All of these statistical analyses in this study were conducted using the *survival* and *pROC* packages in the statistical software R 30.

## Results

### Patients’ characteristics

The first discovery cohort was composed of 81 patients with breast cancer, who underwent complete surgical resection. The surviving patients’ progress was followed up to 11 years. The overall 11‐year OS and DFS rates for all patients in discovery cohort were 86.4% (70 patients) and 76.5% (62 patients), respectively. While the second discovery cohort was composed of 16 breast cancer patients, the surviving patients’ progress was followed up to 3 years. The overall 3‐year OS and DFS rates for all patients in the second discovery cohort were 50.0% (8 patients) and 68.8% (11 patients), respectively (Table 1). Of the patients that died in the discovery cohorts, relapse was the cause of all 11 in the first cohort, but only 4 of the 8 in the second cohort (Table 1).

**Table 1:** OS and DFS rates for all patients in the first and second discovery cohorts

# patients	OS			DFS		
	Dead	Survive	# Probes	Relapse	Nonrelapse	# Probes
First cohort	11	70	9980	19	62	9973
Second cohort	8	8	15,589	5	11	15,102
Combined cohort	19	78	10,337	24	73	10,611

### Genes affecting postoperative OS and DFS

To correct for bias between microarrays, we first applied quantile normalization to all microarray data for each Cy and slide set. The signal ratio of each probe was then calculated according to the following procedure. If both the Cy5 and Cy3 signal of a probe were < 10,000, the ratio was handled as missing value. If only one of the Cy3 or Cy5 signal was < 10,000, the lower signal was converted to 10,000. The ratio was calculated by the Cy5/Cy3 signal intensity. The log_2_‐transformed ratio was converted to a *z*‐score for each microarray slide, and further converted to a *z*‐score for each gene in each data for cross‐platform study (see the Materials and Methods). More than half of probes in both groups (OS: dead and survival, DFS: relapse and nonrelapse) were missing values and were excluded from this study. Of the 27,647 probes, 9980 and 9973 were used to in the final gene selection for postoperative OS and DFS prediction models in the first discovery cohort; 15,589 and 15,102 in the second discovery cohort, respectively (Table 1). We examined probes/genes associated with postoperative OS and DFS using a Cox proportional hazard model with time since diagnosis (see Materials and Methods). As a final result, we detected seven (*LAMB1*,* TMEM189‐UBE2V*,* ESYT1*,* TUBB2A*,* ADAM9*,* JUP*, and *SMARCA2*) and three (*KIAA0196*,* LAMB1*, and *MTMR3*) genes associated with postoperative OS and DFS, respectively (Table 2). The laminin subunit beta 1, encoded by *LAMB1*, was detected as a gene associated with both postoperative OS and DFS (OS: *q* = 0.041, HR = 0.67, 95% CI = 0.52–0.87; DFS: *q* = 0.18, HR = 0.59, 95% CI = 0.44–0.79; Table 2). The microarrays used in the first and second discovery cohorts were not designed with probes for several of the MammaPrint genes, and many others were excluded from the analysis due to low signal intensities and missing values. Of the 70 MammaPrint genes, 37 were included in the prediction model construction (Table S2). We estimated the regression coefficients for these 37 MammaPrint genes in discovery and validation cohorts using a Cox proportional hazard model, and examined these genes’ correlations between the different cohorts using Pearson's correlation test. Pearson's correlation coefficients of 0.50 (*P* = 0.0016) and 0.49 (*P* = 0.0019) were obtained in the GSE42568 validation cohort for OS and DFS, respectively. Similar results were obtained in the GSE1456 validation cohort; Pearson's correlation coefficients of 0.66 (*P* = 1.03 × 10^−3^) and 0.65 (*P* = 1.49 × 10^−5^) for OS and DFS, respectively. These results showed positive correlation between discovery and validation cohorts in both OS and DFS (Table S3).

**Table 2:** Genes affecting postoperative OS and DFS using Cox proportional hazard models

Gene symbol (Gene ID)	(1) *P*‐value in first discovery cohort	(2) Coefficient		(3) *q*‐value in the combined cohort		
		First discovery cohort	Second discovery cohort	*q*‐value	HR	95% CI
OS						
*LAMB1* (3912)	1.27 × 10^−06^	−1.31	−0.075	0.041	0.67	0.52–0.87
*TMEM189‐UBE2V1* (387522)	2.95 × 10^−05^	1.79	0.04	0.034	2.20	1.34–3.60
*ESYT1* (23344)	0.00012	1.30	0.38	0.042	2.02	1.27–3.21
*JUP* (3728)	0.00018	1.01	0.71	0.0012	2.43	1.62–3.64
*SMARCA2* (6595)	0.00020	−1.45	−0.15	0.00046	0.29	0.18–0.48
*TUBB2A* (7280)	0.00022	1.32	0.33	0.045	1.96	1.24–3.10
*ADAM9* (8754)	0.00024	1.1280	0.04675	0.071	1.74	1.16–2.62
DFS						
*KIAA0196* (9897)	5.52 × 10^−05^	0.90	0.067	0.14	2.31	1.55–3.44
*MTMR3* (8897)	6.33 × 10^−05^	−1.04	−0.32	0.17	0.43	0.28–0.67
*LAMB1* (3912)	0.00025	−0.60	−0.42	0.18	0.59	0.44–0.79

We performed gene selection through the following steps: (1) The top significantly differentially expressed genes under different conditions (OS, survival and dead; DFS, relapse and nonrelapse) using a Cox proportional hazard model from the first discovery cohort data (*P* < 0.0003). (2) Genes showing the effects in the same direction as genes detected in step 1 from the second discovery cohort. (3) Genes showing *q* < 0.2 in the combined discovery cohort. HR, hazard ratio.

### Evaluation of postoperative OS and DFS prediction models

**Figure 1:**
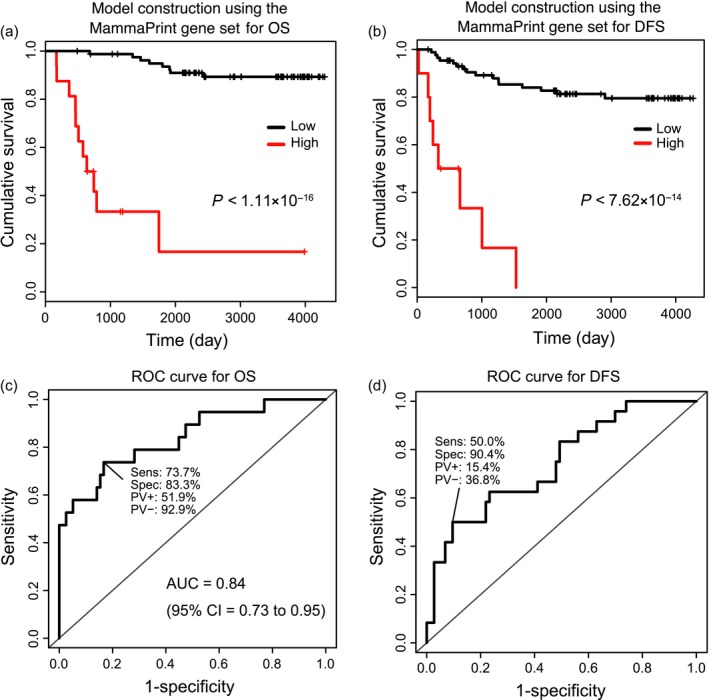
The Kaplan–Meier curves and the receiver operator characteristic curves for the prediction models using the MammaPrint gene set. Based on the MammaPrint gene set, prediction models were constructed from our combined discovery cohort data using a Cox proportional hazard model. A prognostic index was assigned to each subject was calculated by applying the MammaPrint gene set to each of the prediction models. Based on this prognostic index, the optimal cutoff values indicated by a minimum log‐rank trend test *P*‐value were determined by comparing the difference between high‐ (red) and low‐risk (black) groups in the combined discovery cohort for cumulative overall survival (OS) (A) and disease‐free survival (DFS) (B). OS: optimal cutoff = 6.59, minimum *P* < 1.11 × 10^−16^, DFS: optimal cutoff = 8.16, minimum *P* = 7.99 × 10^−15^. The AUC achieved in OS was 0.84 (95% CI = 0.73 to 0.95) (C) and the AUC for DFS was 0.68 (95% CI = 0.56–0.81) (D). The receiver operator characteristic curve achieved a maximum sensitivity of 0.74 and specificity of 0.83 in OS (C), and a maximum sensitivity of 0.54 and specificity of 0.81 in DFS (D).

Based on the MammaPrint gene set, we constructed prediction models for OS and DFS in the combined discovery cohort data using a Cox proportional hazard model. We calculated a prognostic index assigned to each subject by applying the MammaPrint gene set to OS and in DFS for each of the prediction models. Based on the prognostic index, we divided samples of the discovery cohort into high‐ and low‐risk groups. The optimal cutoff values were detected by using the minimum log‐rank trend test *P*‐value and comparing the differences within survival in OS and DFS as determined by KM curves (OS: optimal cutoff = 6.59, minimum *P* < 1.11 × 10^−16^, Fig. 1A; DFS: optimal cutoff = 8.91, minimum *P* < 7.62 × 10^−14^, Fig. 1B). We also performed AUC estimations based on the prognostic index. The AUC achieved in OS was 0.84 (95% CI = 0.73–0.95, Fig. 1C) and the AUC for DFS was 0.74 (95% CI = 0.63–0.86, Fig. 1D). The ROC curve achieved a maximum sensitivity of 0.74 and specificity of 0.83 in OS (Fig. 1C), and a maximum sensitivity of 0.50 and specificity of 0.90 in DFS (Fig. 1D).

**Figure 2:**
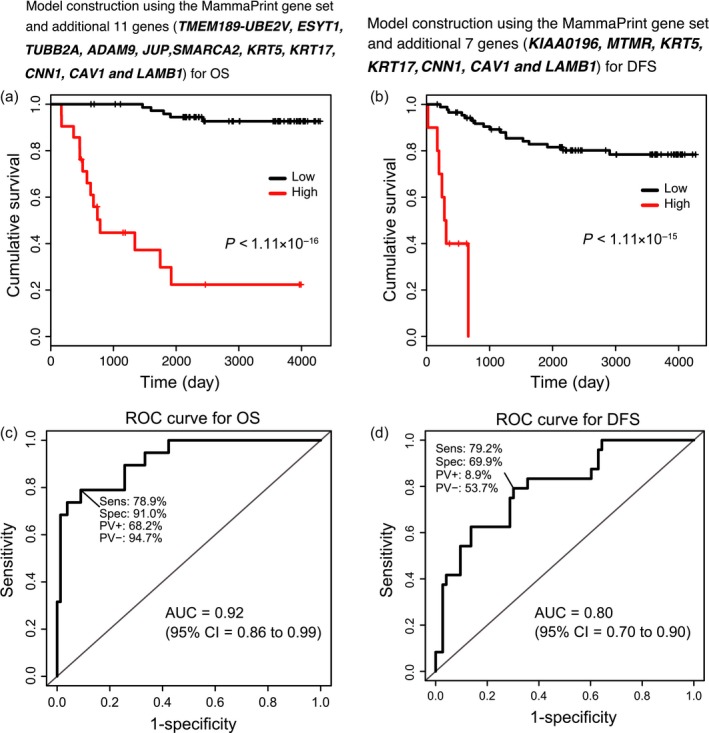
The Kaplan–Meier curves and the receiver operator characteristic curves for the prediction models using our improved gene sets. On the basis of our improved gene sets, prediction models were constructed from our combined discovery cohort data using a Cox proportional hazard model. Prognostic index assigned to each subject was calculated by applying the improvement related genes to each of the prediction models. Based on this prognostic index, optimal cutoff values indicated by a minimum log‐rank trend test *P*‐value were determined by comparing the difference between high‐ (red) and low‐risk (black) groups in the combined discovery cohort for cumulative overall survival (OS) (A) and disease‐free survival (DFS) (B). OS: optimal cutoff = 7.14, minimum *P* < 1.11 × 10^−16^, DFS: optimal cutoff = 9.60, minimum *P* = 7.99 × 10^−15^. The AUC achieved in OS was 0.92 (95% CI = 0.86–0.99) (C) and the AUC for DFS was 0.72 (95% CI = 0.60–0.84) (D). The receiver operator characteristic curve achieved a maximum sensitivity of 0.79 and specificity of 0.91 in OS (C), and a maximum sensitivity of 0.79 and specificity of 0.62 in DFS (D).

In order to further improve the MammaPrint gene set prediction models, we incorporated the seven and three genes detected to be associated with OS and DFS in our analysis (Table 2), and the five genes associated with a poor DFS and OS as reported by Sorlie et al. 14 to the MammaPrint gene set (Table S2), and constructed prediction models in the combined discovery cohort using a Cox proportional hazard model. We calculated a prognostic index assigned to each subject by applying 48 genes (Tables 2 and S2) to OS and 44 genes to DFS (Table 2 and Table S2) for each of the prediction models, and divided the samples of the discovery cohort into high‐ and low‐risk groups. The optimal cutoff values were detected using the minimum log‐rank trend test *P*‐value when comparing the differences within survival in OS and DFS as determined by KM curves (OS: optimal cutoff = 7.14, minimum *P* < 1.11 × 10^−16^, Figure 2A, DFS: optimal cutoff = 6.90, minimum *P* < 1.11 × 10^−15^, Fig. 2B). We also performed AUC estimations based on the prognostic index. The AUC achieved in OS was 0.92 (95% CI = 0.86–0.99, Fig. 2C) and the AUC for DFS was 0.80 (95% CI = 0.70–0.90, Fig. 2D). The ROC curve achieved a maximum sensitivity of 0.79 and specificity of 0.91 in OS (Fig. 2C), and a maximum sensitivity of 0.79 and specificity of 0.70 in DFS (Fig. 2D).

### Validation of our prediction models using independent test sets

**Figure 3:**
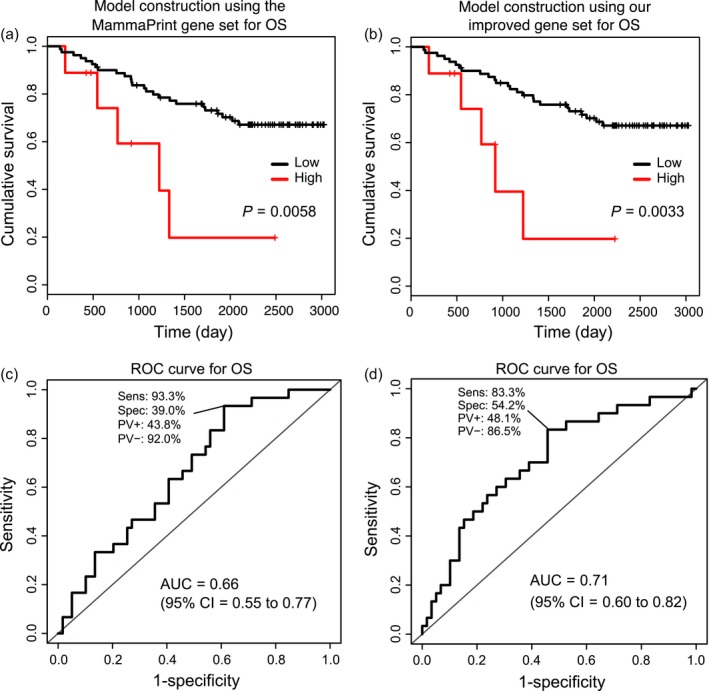
Verification our prediction models using the GSE42568 cohort (validation cohort) obtained from the GEO database in OS. Kaplan–Meier curves for the OS prediction models using the MammaPrint gene set (A) and using our OS improved gene set (B) when using GSE42568's data for the risk prediction model verification. Receiver operator characteristic curves for the OS prediction models using the MammaPrint gene set (C) and using our OS improved gene set (D) when using GSE42568's data for the risk prediction model verification.

To ensure the generality of our prediction models, we applied our models to an additional three independent test datasets (validation cohorts). The independent validation sets were obtained from the GEO and TCGA databases, and consisted of 104 patients (accession GSE42568 24), 159 patients (accession GSE1456 25), and 1090 patients (TCGA) with breast cancer. First, we examined whether prediction models using the MammaPrint gene set and the OS improved gene set were validated in the independent test sets for OS. We calculated a prognostic index for each subject by applying the MammaPrint gene set and the OS improved gene set to each of the prediction models. Using the optimal cutoff values predetermined in the discovery cohort (MammaPrint gene set = 6.59, OS improved gene set = 7.14), the samples of the independent test dataset were divided into high‐ and low‐risk groups. The difference within survival in OS was determined by KM curves, showing statistically significant difference for both the prediction models using the MammaPrint gene set (log‐rank trend *P* = 0.0058, HR = 0.27, 95% CI = 0.10–0.73, Fig. 3A) and using the OS improved gene set (log‐rank trend *P* = 0.0033, HR = 0.25, 95% CI = 0.094–0.68, Fig. 3B) in the GSE42568 cohort. Similar results were also obtained in the GSE1456 cohort, showing a statistically significant difference for both the prediction models using the MammaPrint gene set (log‐rank trend *P* = 0.0061, HR = 0.35, 95% CI = 0.16–0.77, Fig. S1A) and using the OS improved gene set (log‐rank trend *P* = 0.00058, HR = 0.29, 95% CI = 0.14–0.62, Fig. S1B). In addition, similar results were obtained in TCGA cohort, showing a statistically significant difference for the prediction model using the OS improved gene set (log‐rank trend *P* = 0.035, HR = 0.66, 95% CI = 0.45–0. 97, Fig. S2B), although the model using the MammaPrint gene set did not show a statistically significant difference (log‐rank trend *P* = 0.41, HR = 1.20, 95% CI = 0.78–1.83, Fig. S2A). The AUC achieved in the prediction model using the MammaPrint gene set was 0.66 (95% CI = 0.55–0.77, Fig. 3C) in GSE42568, 0.76 (95% CI = 0.67–0.85, Fig. S1C) in GSE1456, and 0.55 (95% CI = 0.50–0.60, Fig. S2C) in TCGA. The AUC for the OS improved gene set was 0.71 (95% CI = 0.60–0.82, Fig. 3D) in GSE42568, 0.78 (95% CI = 0.69–0.87, Fig. S1D) in GSE1456, and 0.53 (95% CI = 0.47–0.58, Fig. S2D) in TCGA. In OS, an improvement in the risk prediction ability was observed using the OS improved gene set model.

**Figure 4:**
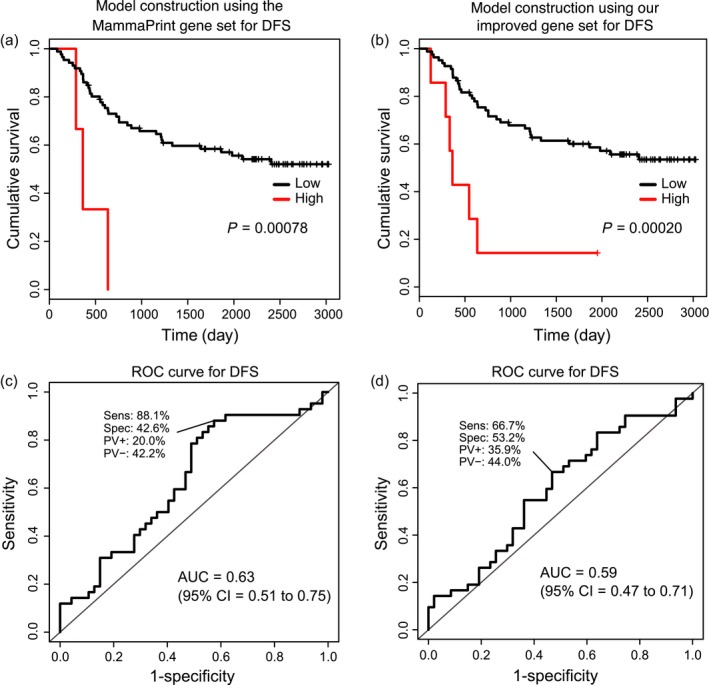
Verification our prediction models using the GSE42568 cohort (validation set) obtained from the GEO database in DFS. Kaplan–Meier curves for the DFS prediction models using the MammaPrint gene set (A) and using our DFS improved gene set (B) when using GSE42568's data for the risk prediction model verification. Receiver operator characteristic curves for the DFS prediction models using the MammaPrint gene set (C) and using our DFS improved gene set (D) when using GSE42568's data for the risk prediction model verification.

In a similar way, for DFS, we compared prediction model using the MammaPrint gene set to that using the DFS improved gene set in the validation cohorts. As described previously, based on the optimal cutoff values predetermined in the discovery cohort (MammaPrint gene set = 8.91, DFS improved gene set = 6.90), we divided the samples of the independent dataset into high‐ and low‐risk groups. The difference within survival in DFS was determined by KM curves. As is the case with the OS, the KM curves showed a statistically significant difference for both the models using the MammaPrint gene set (log‐rank trend *P* = 0.00078, HR = 0.16, 95% CI = 0.05–0.54, Fig. 4A) and the DFS improved gene set (log‐rank trend *P* = 0.00020, HR = 0.22, 95% CI = 0.089–0.52, Fig. 4B) in the GSE42568 cohort. Similar results were obtained in the GSE1456 cohort, showing a statistically significant difference for the prediction model using the DFS improved gene set (log‐rank trend *P* = 0.0075, HR = 0.36, 95% CI = 0.17–0.79, Fig. S3B). The model using the MammaPrint gene set did not show a statistically significant difference (log‐rank trend *P* = 0.27, HR = 0.52, 95% CI = 0.16–1.70, Fig. S3A). The AUCs achieved for the MammaPrint gene set models was then 0.63 (95% CI = 0.51–0.75, Fig. 4C) in GSE42568 and 0.73 in GSE1456 (95% CI = 0.64–0.82, Fig. S3C). The AUC for that using the DFS improved gene set was 0.59 in GSE42568 (95% CI = 0.47–0.71, Fig. 4D) and 0.72 in GSE1456 (95% CI = 0.63–0.80, Fig. S3D). Similar outcome was observed in DFS for KM curves as in OS. These results suggest that there is still much room for improvement in the current methods for prediction of postoperative OS and DFS in breast cancer patients.

Some traditional clinical markers, such as ER/PR, HER2, node stage, and tumor size, have been approved to affect clinical outcome of breast cancer 31, 32, 33, 34, 35. As we were able to obtain specific phenotypes (ER, HER2, and grade) of breast cancers from a validation cohort (GSE1456 25), we further investigated if our genes (OS: seven genes, DFS: three genes) could predict OS and DFS independently by comparing the specific phenotype with and without our findings, respectively (i.e., deviation from a multiplicative model). As a result, significant *P*‐values were observed for our OS genes (Table S4), although there were no significant *P*‐values in our DFS genes (note that even when the phenotypes were not considered, our DFS genes did not result in significant *P*‐values). These results showed that our OS genes could predict OS independently. While it is still unclear whether or not our DFS genes alone could predict DFS independently, this DFS improved gene set was both validated using several independent cohorts, and superior the results using the MammaPrint gene set. These results showed that our DFS improved gene set contributed to an improvement to the prediction models using only the MammaPrint gene set.

We also examined the efficacy of the prediction models using the improved gene sets excluding the MamaPrint gene set. We calculated a prognostic index assigned to each subject by applying 11 genes to OS and 7 genes to DFS for each of the prediction models (Tables 2 and S2), and divided the samples of the discovery cohort into high‐ and low‐risk groups. The optimal cutoff values were detected using the minimum log‐rank trend test *P*‐value when comparing the differences within survival in OS and DFS as determined by KM curves (OS: optimal cutoff = 3.46, minimum *P* = 1.11 × 10^−15^, DFS: optimal cutoff = 2.07, minimum *P* = 5.88 × 10^−15^). On the basis of these optimal cutoff values determined in the discovery cohort, we then divided the samples of the independent dataset into high‐ and low‐risk groups. The differences within survival in OS and DFS were determined by KM curves. However, the KM curves did not show a statistically significant difference for both models (OS: log‐rank trend *P* = 0.12, HR = 0.48, 95% CI = 0.19–1.23, Fig. S4A; DFS: log‐rank trend *P* = 0.057, HR = 0.47, 95% CI = 0.21–1.04, Fig. S4B). The AUCs achieved in the OS and DFS models were 0.65 (95% CI = 0.53–0.77, Fig. S4C) and 0.51 (95% CI = 0.40–0.63, Fig. S4D), respectively.

## Discussion

Despite complete surgical resection of the breast cancer, clinical outcome after surgical operation varies greatly among patients. This suggests that certain genes may play a key role in the prognosis of breast cancer, and the observed variation could be due to gene expression. Previous studies have reported several differentially expressed genes associated with OS and DFS using microarray expression data 14, and several genetic tests using gene expression profiles have been developed to predict clinical outcomes 17, 18. One of the tests, MammaPrint 19, successfully classifies tumors into two groups (good prognosis and poor prognosis) for OS and DFS 20. However, subsequent improvement of these prediction models has yet to be reported.

In order to identify additional genes that could improve current prediction models of OS and DFS, we investigated the gene expression data of breast cancer patients using a Cox proportional hazard model. We detected seven and three genes associated with postoperative OS and DFS, respectively. One gene, *LAMB1*, was common to both OS and DFS gene sets, suggesting that this gene may be related to not only the sensitivity to the adjuvant therapy, but also to the malignant potential of the breast cancer cells. A previous study reported that overexpression of *LAMB1* is associated with both poor OS and DFS in breast cancer 14. In addition, Yunchao et al. reported that Ubiquitin1, encoded by *UBQLN1*, is associated with poor prognosis in breast cancer 36. As this gene interacts with ubiquitin conjugating enzyme E2 variant 1 (encoded by *UBE2V1*, which was detected as a gene associated with OS, Table 2), this *UBE2V1* seems to be a novel molecular biomarker able to predict poor prognosis in breast cancer. While the other genes also have a possibility to be novel biomarkers, we did not find any additional supporting evidence in the current literature. Further functional analysis of the above genes might clarify the biological mechanism regarding the regulation of the prognosis of the patients with breast cancer.

We examined whether prediction models using the improved gene sets contributed to an improvement of the prediction models using only the MammaPrint gene set. To evaluate the effectiveness of our prediction models, we used KM curves and AUCs. Further verification using independent datasets (validation cohorts) was necessary to validate the effectiveness of our prediction models and to show that our models were not overfit to our training data. We validated the effectiveness of our models using several independent data obtained from the GEO and TCGA databases. This result suggests that there is still room for improvement for predicting postoperative OS and DFS in breast cancer, although it may be necessary to compare the models using our improved gene sets to those using the full set of MammaPrint genes for clinical use, as the expression of many were not available in our discovery cohort data and the validation cohort data obtained from the GEO and TCGA databases. In addition, cancer treatment and the response to treatment directly influence the subsequent outcome of the disease 37, 38. Therefore, it could be suggested that our improved gene sets influence the outcome of specific breast cancer treatments rather than overall outcome. Further investigation using response information to specific therapies should be required in the future.

Steroid hormone receptors (HRs; i.e., estrogen receptor or progesterone receptor) are important prognostic and predictive factors for response to endocrine therapy in the treatment of breast cancer 31. The hormone receptor‐positive tumors (HR‐positive) generally have a favorable prognosis 32. In addition, human epidermal growth factor receptor 2 (HER2) overexpression (HER2‐positive) is also associated with worse clinical outcome (worse prognosis) of breast cancer 33, 34. It has been statistically shown that the 10‐year prognosis of patients is worse than that of those with HER2‐negative 35. Although we could not obtain the information in this study, we believe that prediction models considering the HR and HER2 status could contribute to further improvements of our models. On the other hand, the development of next‐generation sequencing technology 39, 40, 41 has facilitated the cost‐effective comprehensive analysis of gene expression by RNA‐seq. In the future, we believe that further replication of this analysis using larger sample sizes will lead to a greater improvement in the performance of our prediction models for its practical clinical use.

## Conflict of Interest

The authors declare no competing financial interests.

## Supporting information


**Table S1.** First and second discovery cohorts’ clinical information.
**Table S2.** 37 MammaPrint genes and five genes associated with a poor OS and DFS used for prediction model construction.
**Table S3.** Regression coefficients of 37 MammaPrint genes using a Cox proportional hazard model in discovery cohort and validation cohorts.
**Table S4.** Independent OS and DFS predictions by our genes.
**Figure S1.** Verification of our prediction models using GSE1456 cohort (validation cohort) obtained from the GEO database in OS.
**Figure S2.** Verification of our prediction models using TCGA cohort (validation cohort) in OS.
**Figure S3.** Verification of our prediction models using GSE1456 cohort (validation set) obtained from the GEO database in DFS.
**Figure S4.** Verification of our prediction models without MammaPrint gene sets using an independent test set (validation set) obtained from GEO database.
