# Supplementary material for: The prediction models for postoperative overall survival and disease‐free survival in patients with breast cancer
Source: Cancer Med. 2017 May 24;6(7):1627–38. doi: 10.1002/cam4.1092 (PMC5504310; doi:10.1002/cam4.1092)
Supplement: Supplementary file 1 — Table S1. First and second discovery cohorts’ clinical information. Table S2. 37 MammaPrint genes and five genes associated with a poor OS and DFS used for prediction model construction. Table S3. Regression coefficients of 37 MammaPrint genes using a Cox proportional hazard model in discovery cohort and validation cohorts. Table S4. Independent OS and DFS predictions by our genes. Figure S1. Verification of our prediction models using GSE1456 cohort (validation cohort) obtained from the GEO database in OS. Figure S2. Verification of our prediction models using TCGA cohort (validation cohort) in OS. Figure S3. Verification of our prediction models using GSE1456 cohort (validation set) obtained from the GEO database in DFS. Figure S4. Verification of our prediction models without MammaPrint gene sets using an independent test set (validation set) obtained from GEO database. [file CAM4-6-1627-s001.docx]

**Supplementary Information**

**The prediction models for postoperative overall survival and disease-free survival in patients with breast cancer**

Daichi Shigemizu^1,2,3^, Takuji Iwase^4^, Masataka Yoshimoto^5^, Yasuyo Suzuki^6^, Fuyuki Miya^1,2^, Keith A Boroevich^2^, Toyomasa Katagiri^7,8^, Hitoshi Zembutsu^9†^**,** and Tatsuhiko Tsunoda^1,2,3†^

1. Department of Medical Science Mathematics, Medical Research Institute, Tokyo Medical and Dental University, Tokyo, Japan.
2. Laboratory for Medical Science Mathematics, RIKEN Center for Integrative Medical Sciences, Yokohama, Japan.
3. CREST, Japan Science and Technology Agency, Tokyo, Japan.
4. Department of Breast Surgical Oncology, Breast Oncology Center, Cancer Institute Hospital, Japanese Foundation for Cancer Research, Tokyo, Japan.
5. Yoshimoto Breast Clinic, Tokyo, Japan.
6. 1st Department of Surgery, Sapporo Medical University, School of Medicine, Sapporo, Japan.
7. Division of Genome Medicine, Institute for Genome Research, University of Tokushima, Tokushima, Japan.
8. Human Genome Center, Institute of Medical Science, The University of Tokyo, Tokyo, Japan.
9. Division of Genetics, National Cancer Center, Research Institute, Tokyo, Japan.

^†^These authors should be considered co-corresponding authors.

†Address all correspondence to:

Tatsuhiko Tsunoda, Ph.D.(Medicine) & Ph.D.(Eng.)

Professor, Department of Medical Science Mathematics,

Medical Research Institute, Tokyo Medical and Dental University.

1-5-45 Yushima, Bunkyo-ku, Tokyo 113-8510, Japan.

Phone: +81-3-5803-4175

E-mail: [tsunoda.mesm@mri.tmd.ac.jp](mailto:tsunoda.mesm@mri.tmd.ac.jp)

**Table S1. First and second cohorts’ clinical information**

| Cohort | Id | (a) Date of surgery | (b) Date of recurrence | (c) Date of death | (d) Prognosis date confirmed | DFS (day) | OS (day) |
| --- | --- | --- | --- | --- | --- | --- | --- |
| First | 1 | 2000.1.6 | 2004.7.12 |  | 2011.12.13 | 1626 | 4297 |
|  | 2 | 2000.1.6 |  |  | 2011.3.11 | 4025 | 4025 |
|  | 3 | 2000.1.7 | 2003.7.8 |  | 2011.9.8 | 1261 | 4201 |
|  | 4 | 2000.1.13 |  |  | 2007.12.5 | 2842 | 2842 |
|  | 5 | 2000.1.14 |  |  | 2006.6.1 | 2297 | 2297 |
|  | 6 | 2000.1.19 |  |  | 2009.11.26 | 3547 | 3547 |
|  | 7 | 2000.1.21 | 2000.8.29 | 2001.12.12 | 2001.12.12 | 281 | 681 |
|  | 8 | 2000.1.26 |  |  | 2011.12.5 | 4269 | 4269 |
|  | 9 | 2000.1.28 |  |  | 2006.6.5 | 2287 | 2287 |
|  | 10 | 2000.2.23 |  |  | 2011.3.11 | 3978 | 3978 |
|  | 11 | 2000.3.2 |  |  | 2011.4.4 | 3992 | 3992 |
|  | 12 | 2000.3.6 |  |  | 2007.1.12 | 2466 | 2466 |
|  | 13 | 2000.3.15 |  |  | 2011.3.22 | 3967 | 3967 |
|  | 14 | 2000.3.22 |  |  | 2010.4.18 | 3626 | 3626 |
|  | 15 | 2000.3.22 |  |  | 2011.12.14 | 4222 | 4222 |
|  | 16 | 2000.3.27 |  |  | 2011.7.6 | 4059 | 4059 |
|  | 17 | 2000.3.28 |  |  | 2010.3.16 | 3588 | 3588 |
|  | 18 | 2000.3.29 |  |  | 2011.8.16 | 4097 | 4097 |
|  | 19 | 2000.4.19 |  |  | 2011.4.16 | 3957 | 3957 |
|  | 20 | 2000.4.19 |  |  | 2006.3.6 | 2117 | 2117 |
|  | 21 | 2000.5.8 | 2003.2.21 |  | 2007.3.8 | 1003 | 2460 |
|  | 22 | 2000.5.15 |  |  | 2011.7.22 | 4027 | 4027 |
|  | 23 | 2000.6.1 | 2002.4.3 | 2005.4.7 | 2005.4.7 | 662 | 1746 |
|  | 24 | 2000.6.5 |  |  | 2011.6.7 | 3962 | 3962 |
|  | 25 | 2000.6.5 | 2002.2.12 | 2005.10.6 | 2005.10.6 | 607 | 1921 |
|  | 26 | 2000.6.28 | 2001.1.15 | 2002.7.22 | 2002.7.22 | 197 | 744 |
|  | 27 | 2000.6.28 |  |  | 2011.9.26 | 4048 | 4048 |
|  | 28 | 2000.7.10 |  |  | 2008.11.25 | 3015 | 3015 |
|  | 29 | 2000.7.10 |  |  | 2011.10.20 | 4060 | 4060 |
|  | 30 | 2000.7.17 |  |  | 2010.8.17 | 3630 | 3630 |
|  | 31 | 2000.8.23 |  |  | 2010.12.24 | 3721 | 3721 |
|  | 32 | 2000.8.30 | 2008.9.25 |  | 2008.10.1 | 2905 | 2911 |
|  | 33 | 2000.9.1 |  |  | 2011.8.2 | 3931 | 3931 |
|  | 34 | 2000.9.11 |  |  | 2011.10.7 | 3986 | 3986 |
|  | 35 | 2000.9.20 | 2003.3.25 | 2006.1.9 | 2006.1.9 | 905 | 1909 |
|  | 36 | 2000.9.20 |  |  | 2011.10.16 | 3986 | 3986 |
|  | 37 | 2000.9.22 | 2006.1.19 |  | 2011.12.5 | 1917 | 4033 |
|  | 38 | 2000.10.2 |  |  | 2010.11.18 | 3646 | 3646 |
|  | 39 | 2000.10.3 |  |  | 2010.10.28 | 3625 | 3625 |
|  | 40 | 2000.10.5 |  |  | 2010.10.27 | 3622 | 3622 |
|  | 41 | 2000.10.6 |  |  | 2007.2.16 | 2290 | 2290 |
|  | 42 | 2000.10.11 |  |  | 2007.6.18 | 2407 | 2407 |
|  | 43 | 2000.10.18 |  |  | 2006.12.18 | 2220 | 2220 |
|  | 44 | 2000.11.2 |  |  | 2010.12.9 | 3637 | 3637 |
|  | 45 | 2000.11.2 |  |  | 2006.12.18 | 2206 | 2206 |
|  | 46 | 2000.11.13 | 2000.12.5 | 2003.1.20 | 2003.1.20 | 22 | 787 |
|  | 47 | 2000.11.20 |  |  | 2011.1.13 | 3653 | 3653 |
|  | 48 | 2000.11.30 |  |  | 2011.10.21 | 3921 | 3921 |
|  | 49 | 2000.12.6 | 2001.11.26 | 2005.5.15 | 2005.5.15 | 350 | 1599 |
|  | 50 | 2000.12.6 |  |  | 2011.9.21 | 3885 | 3885 |
|  | 51 | 2000.12.7 |  |  | 2006.11.30 | 2153 | 2153 |
|  | 52 | 2000.12.8 |  |  | 2006.11.30 | 2152 | 2152 |
|  | 53 | 2000.12.13 |  |  | 2006.12.18 | 2165 | 2165 |
|  | 54 | 2000.12.13 | 2004.3.16 | 2007.9.9 | 2007.9.9 | 1173 | 2426 |
|  | 55 | 2000.12.14 |  |  | 2012.1.4 | 3980 | 3980 |
|  | 56 | 2000.12.25 |  |  | 2011.1.29 | 3634 | 3634 |
|  | 57 | 2000.12.27 |  |  | 2006.12.5 | 2138 | 2138 |
|  | 58 | 2000.12.28 |  |  | 2011.12.29 | 3961 | 3961 |
|  | 59 | 2001.1.18 |  |  | 2011.1.6 | 3588 | 3588 |
|  | 60 | 2001.1.19 |  |  | 2006.11.30 | 2111 | 2111 |
|  | 61 | 2001.1.26 | 2003.3.14 | 2005.2.20 | 2005.2.20 | 768 | 1464 |
|  | 62 | 2001.2.5 |  |  | 2011.3.24 | 3649 | 3649 |
|  | 63 | 2001.2.14 |  |  | 2011.1.20 | 3576 | 3576 |
|  | 64 | 2001.2.14 |  |  | 2011.10.4 | 3830 | 3830 |
|  | 65 | 2001.2.15 |  |  | 2011.3.24 | 3639 | 3639 |
|  | 66 | 2001.2.19 |  |  | 2007.12.3 | 2444 | 2444 |
|  | 67 | 2001.2.20 | 2005.5.20 |  | 2011.9.22 | 1530 | 3812 |
|  | 68 | 2001.2.22 |  |  | 2011.3.31 | 3639 | 3639 |
|  | 69 | 2001.2.23 |  |  | 2011.4.26 | 3663 | 3633 |
|  | 70 | 2001.2.23 |  |  | 2007.9.12 | 2359 | 2359 |
|  | 71 | 2001.3.1 |  |  | 2011.12.14 | 3883 | 3883 |
|  | 72 | 2001.3.14 |  |  | 2011.4.7 | 3623 | 3623 |
|  | 73 | 2001.3.21 |  |  | 2009.7.30 | 3009 | 3009 |
|  | 74 | 2001.3.26 | 2004.9.24 |  | 2011.9.30 | 1258 | 3784 |
|  | 75 | 2001.3.30 |  |  | 2011.3.1 | 3571 | 3571 |
|  | 76 | 2001.4.5 | 2003.4.4 | 2004.12.26 | 2004.12.26 | 719 | 1341 |
|  | 77 | 2001.4.6 |  |  | 2007.9.14 | 2318 | 2318 |
|  | 78 | 2001.4.25 |  |  | 2011.4.7 | 3582 | 3582 |
|  | 79 | 2000.8.2 |  |  | 2007.2.26 | 2364 | 2364 |
|  | 80 | 2000.8.16 | 2006.8.17 |  | 2008.8.29 | 2161 | 2893 |
|  | 81 | 2000.12.20 | 2002.7.9 | 2005.12.20 | 2005.12.20 | 559 | 1800 |
| Second | 1 | 2003.2.4 | 2003.12.15 | 2004.9.11 | 2004.9.11 | 311 | 577 |
|  | 2 | 2003.2.14 | 2003.11.27 |  | 2006.5.31 | 283 | 1187 |
|  | 3 | 2003.2.19 |  |  | 2006.5.2 | 1153 | 1153 |
|  | 4 | 2003.4.15 | 2004.3.12 |  | 2006.5.16 | 327 | 1111 |
|  | 5 | 2003.5.20 |  | 2004.10.18 | 2004.10.18 | 508 | 508 |
|  | 6 | 2003.6.3 |  | 2004.6.7 | 2004.6.7 | 364 | 364 |
|  | 7 | 2003.6.24 |  |  | 2004.11.12 | 488 | 488 |
|  | 8 | 2003.7.22 |  |  | 2006.5.30 | 1028 | 1028 |
|  | 9 | 2003.10.14 |  | 2005.7.20 | 2005.7.20 | 636 | 636 |
|  | 10 | 2004.5.18 | 2005.1.22 | 2005.8.30 | 2005.8.30 | 244 | 462 |
|  | 11 | 2004.6.3 |  |  | 2006.6.22 | 739 | 739 |
|  | 12 | 2004.7.20 |  |  | 2006.6.22 | 692 | 692 |
|  | 13 | 2004.7.20 |  |  | 2006.5.1 | 641 | 641 |
|  | 14 | 2004.8.17 |  | 2005.2.6 | 2005.2.6 | 169 | 169 |
|  | 15 | 2004.8.17 |  | 2005.12.1 | 2005.12.1 | 464 | 464 |
|  | 16 | 2005.3.9 | 2005.8.29 | 2005.9.1 | 2005.9.1 | 170 | 172 |

**Table S2. 37 MammaPrint genes and 5 genes associated with a poor OS and DFS used for prediction model construction**

| **Category** | **Gene name** | **Gene description** |
| --- | --- | --- |
| MammaPrint gene set | *EGLN1* | Egl nine homolog 1 (C. elegans) |
|  | *TGFB3* | Transforming growth factor, beta 3 |
|  | *IGFBP5* | Insulin-like growth factor binding protein 5 |
|  | *SCUBE2* | Signal peptide, CUB domain, EGF-like 2 |
|  | *FLT1* | Fms-related tyrosine kinase 1 |
|  | *HRASLS* | HRAS-like suppressor |
|  | *STK32B* | Serine/threonine kinase 32B |
|  | *RASSF7* | Ras association domain family 7 |
|  | *DCK* | Deoxycytidine kinase |
|  | *EXT1* | Exostoses 1 |
|  | *GNAZ* | Guanine nucleotide binding protein (G protein), alpha z polypeptide |
|  | *MTDH* | metadherin |
|  | *PITRM1* | Pitrilysin metalloproteinase 1 |
|  | *QSCN6L1* | Quiescin Q6-like 1 |
|  | *ECT2* | Epithelial cell transforming sequence 2 oncogene |
|  | *KNTC2* | Kinetochore associated 2 |
|  | *MCM6* | MCM6 minichromosome maintenance deficient 6 |
|  | *NUSAP1* | Nucleolar and spindle associated protein 1 |
|  | *ORC6L* | Origin recognition complex, subunit 6 homolog-like |
|  | *TSPYL5* | TSPY-like 5 |
|  | *RUNDC1* | RUN domain containing 1 |
|  | *PRC1* | Protein regulator of cytokinesis 1 |
|  | *RFC4* | Replication factor C 4 |
|  | *DTL* | Denticleless homolog |
|  | *COL4A2* | Collagen, type IV, alpha 2 |
|  | *GPR180* | G protein-coupled receptor 180 |
|  | *GPR126* | G protein-coupled receptor 126 |
|  | *RTN4RL1* | Reticulon 4 receptor-like 1 |
|  | *CDC42BPA* | CDC42 binding protein kinase alpha |
|  | *PALM2* | Paralemmin 2 |
|  | *ALDH4A1* | Aldehyde dehydrogenase 4 family, member A1 |
|  | *PECI* | Peroxisomal D3,D2-enoyl-CoA isomerase |
|  | *GMPS* | Guanine monphosphate synthetase |
|  | *SLC2A3* | Solute carrier family 2, member 3 |
|  | *ZNF533* | Zinc finger protein 533 |
|  | *SERF1A* | Small EDRK-rich factor 1A |
|  | *RAB6B* | RAB6B, member RAS oncogene family |
| Gene set associated with a poor OS and DFS | *KRT5* | Keratin 5 |
|  | *KRT17* | Keratin 17 |
|  | *CNN1* | Calponin 1 |
|  | *CAV1* | Caveolin 1 |
|  | *LAMB1* | Laminin subunit beta 1 |

**Table S3. Regression coefficients of 37 MammaPrint genes using a Cox proportional hazard model in discovery cohort and validation cohorts.**

|  | **OS (coefficient)** | | |  | **DFS (coefficient)** | | |
| --- | --- | --- | --- | --- | --- | --- | --- |
| **Cohort** | **Discovery** | **Validation** |  |  | **Discovery** | **Validation** |  |
| **Gene name** |  | **GSE42568** | **GSE1456** | **TCGA** |  | **GSE42568** | **GSE1456** |
| *EGLN1* | -0.598 | 0.1404 | 0.3671 | 0.04607 | -0.1606 | 0.1951 | 0.3192 |
| *TGFB3* | -0.2342 | -0.3685 | -0.5767 | -0.07538 | -0.2326 | -0.2916 | -0.5283 |
| *IGFBP5* | -0.2783 | 0.04357 | -0.06409 | 0.1521 | 0.1221 | -0.01783 | 0.06526 |
| *SCUBE2* | -0.7224 | -0.5831 | -0.4486 | -0.20214 | -0.6367 | -0.4611 | -0.2955 |
| *FLT1* | -0.1464 | 0.2238 | 0.2349 | 0.2207 | -0.262 | 0.07449 | 0.1534 |
| *HRASLS* | 0.412 | 0.5011 | 0.6039 | 0.05031 | 0.2723 | 0.2967 | 0.5011 |
| *STK32B* | -0.7319 | -0.2874 | -0.4141 | 0.05742 | -0.4403 | -0.3038 | -0.2719 |
| *RASSF7* | 0.5837 | 0.2042 | 0.3945 | -0.27132 | 0.05739 | 0.1035 | 0.1035 |
| *DCK* | -0.01153 | -0.2075 | -0.1188 | -0.05806 | 0.4126 | -0.0571 | 0.4022 |
| *EXT1* | -1.0543 | 0.5825 | -0.2387 | 0.08857 | -0.3046 | 0.4465 | -0.3280 |
| *GNAZ* | 0.2365 | 0.4069 | -0.05077 | 0.12561 | -0.03428 | 0.3289 | -0.06282 |
| *MTDH* | 0.6211 | -0.0622 | 0.4203 | 0.09996 | 0.5136 | 0.1274 | 0.393 |
| *PITRM1* | 0.505 | 0.2759 | 0.4942 | 0.1093 | 0.6047 | 0.2251 | 0.4177 |
| *QSCN6L1* | 0.06413 | 0.07106 | 0.0001225 | - | 0.02704 | 0.105 | 0.0403 |
| *ECT2* | 0.4896 | 0.07654 | 0.3947 | -0.19389 | 0.6336 | -0.05951 | 0.3382 |
| *KNTC2* | -0.4179 | 0.4758 | 0.5313 | - | -0.289 | 0.3282 | 0.4367 |
| *MCM6* | 0.2838 | 0.5526 | 0.4887 | -0.14253 | 0.1514 | 0.3687 | 0.4854 |
| *NUSAP1* | 1.0619 | 0.7128 | 0.6553 | -0.23184 | 0.7917 | 0.3166 | 0.4857 |
| *ORC6L* | 0.05824 | 0.4535 | 0.5276 | - | -0.05777 | 0.345 | 0.5151 |
| *TSPYL5* | 0.06042 | 0.5852 | -0.01134 | -0.03491 | 0.2283 | 0.5039 | -0.2270 |
| *RUNDC1* | -0.3005 | -0.7404 | -0.2985 | -0.07303 | -0.26 | -0.5867 | -0.2513 |
| *PRC1* | 0.8657 | 0.3012 | 0.7186 | -0.18338 | 0.5395 | 0.1832 | 0.6089 |
| *RFC4* | 0.601 | 0.1934 | 0.2303 | -0.1674 | 0.3619 | 0.1354 | 0.2040 |
| *DTL* | 0.6141 | 0.5923 | 0.7644 | -0.22178 | 0.6125 | 0.3658 | 0.6361 |
| *COL4A2* | 0.2324 | 0.1494 | -0.04822 | 0.06637 | 0.1892 | -0.01529 | 0.03635 |
| *GPR180* | 0.8359 | 0.2037 | 0.4868 | 0.18716 | 0.3853 | 0.3237 | 0.3849 |
| *GPR126* | 0.3179 | 0.2133 | 0.2674 | - | 0.08749 | 0.1916 | 0.2393 |
| *RTN4RL1* | -0.3752 | -0.1484 | -0.3263 | -0.1484 | 0.1228 | -0.1943 | -0.1643 |
| *CDC42BPA* | -0.116 | 0.2355 | 0.1999 | 0.26773 | -0.1066 | 0.1466 | 0.1083 |
| *PALM2* | 0.2515 | 0.2221 | - | -0.08935 | 0.3257 | 0.1912 | - |
| *ALDH4A1* | 0.03317 | 0.116 | -0.08731 | 0.18617 | 0.1697 | 0.04155 | -0.1505 |
| *PECI* | -0.2864 | -0.5953 | -0.3144 | - | -0.09856 | -0.5015 | -0.3147 |
| *GMPS* | 0.7439 | 0.5637 | 0.5228 | -0.06679 | 0.5663 | 0.3926 | 0.3844 |
| *SLC2A3* | 0.2243 | 0.06828 | -0.01792 | 0.08234 | -0.03858 | -0.04443 | -0.08165 |
| *ZNF533* | -0.6474 | -0.3977 | -0.3447 | - | -0.3969 | -0.4082 | -0.4204 |
| *SERF1A* | -0.239 | -0.1421 | 0.08351 | 0.05567 | 0.02779 | -0.123 | 0.01175 |
| *RAB6B* | 0.8211 | 0.4573 | -0.1837 | 0.24848 | 0.3298 | 0.319 | -0.1765 |

**Table S4. Independent OS and DFS predictions by our genes**

|  | OS (p-value) | DFS (p-value) |
| --- | --- | --- |
| Our genes (OS = 7 genes, DFS = 3 genes) | 1.08x10^-5^ | 0.198 |
| (ER) vs. (ER + our genes) | 0.0022 | 0.380 |
| (HER2) vs. (HER2 + our genes) | 0.0039 | 0.468 |
| (Grade) vs. (Grade + our genes) | 0.0028 | 0.537 |
| (ER + HER2) vs. (ER + HER2 + our genes) | 0.0139 | 0.372 |
| (ER + Grade) vs. (ER + Grade + our genes) | 0.0264 | 0.558 |
| (HER2 + Grade) vs. (HER2 + Grade +our genes) | 0.0126 | 0.508 |
| (ER + HER2 + Grade) vs. (ER + HER2 + Grade + our genes) | 0.0345 | 0.488 |

**Figure S1. Verification our prediction models using GSE1456 cohort (validation cohort) obtained from the GEO database in OS.**

Kaplan-Meier curves for the OS prediction models using the MammaPrint gene set (a) and using our OS improved gene set (b) when using GSE1456’s data for the risk prediction model verification. ROC curves for the OS prediction models using the MammaPrint gene set (c) and using our OS improved gene set (d) when using GSE1456’s data for the risk prediction model verification.

**Figure S2. Verification our prediction models using TCGA cohort (validation cohort) in OS.**

Kaplan-Meier curves for the OS prediction models using the MammaPrint gene set (a) and using our OS improved gene set (b) when using TCGA’s data for the risk prediction model verification. ROC curves for the OS prediction models using the MammaPrint gene set (c) and using our OS improved gene set (d) when using TCGA’s data for the risk prediction model verification.

**Figure S3. Verification our prediction models using GSE1456 cohort (validation set) obtained from the GEO database in DFS.**

Kaplan-Meier curves for the DFS prediction models using the MammaPrint gene set (a) and using our DFS improved gene set (b) when using GSE1456’s data for the risk prediction model verification. ROC curves for the DFS prediction models using the MammaPrint gene set (c) and using our DFS improved gene set (d) when using GSE1456’s data for the risk prediction model verification.

**Figure S4. Verification our prediction models without MammaPrint gene sets using an independent test set (validation set) obtained from GEO database**
